# A Sorbent with Synthetic Ligand for Removing Pro-atherogenic and Pro-inflammatory Components from Human Blood Plasma

**DOI:** 10.32607/actanaturae.11292

**Published:** 2021

**Authors:** O. A. Dmitrieva, E. D. Ovchinnikova, E. A. Utkina, P. A. Levashov, O. I. Afanasieva, I. Y. Adamova, S. N. Pokrovsky

**Affiliations:** Federal State Budgetary Institution «National Medical Research Center of Cardiology» Ministry of Health of the Russian Federation, Moscow, 121552 Russia; Lomonosov Moscow State University, Moscow, 119991 Russia

**Keywords:** C-reactive protein, atherogenic lipoproteins, lipoprotein (a), atherosclerosis, therapeutic apheresis

## Abstract

Elevated levels of apoB-100 containing lipoproteins and markers of systemic inflammation are often observed in patients with cardiovascular diseases. The concentrations can be reduced by pharmacotherapy or extracorporeal treatment. The sorbent, which removes CRP and atherogenic lipoproteins, simultaneously reduces the bloodstream concentration of these components. The efficacy and selectivity of the designed sorbent were studied, desorption constants of CRP (K_d_ = 4.2 × 10^-8^ M) and LDL (K_d_ = 7.7 × 10^-7^ M) were distribution coefficients of CRP (K_c_ = 101) and Lp(a) (K_c_ = 38) were calculated, and the ability to bind large amounts of atherogenic lipoproteins (up to 32 mg of TC per mL of the sorbent gel) was demonstrated. Our sorbent can be recommended for performing complex removal of CRP and atherogenic lipoproteins from the blood plasma in patients with refractory hyperlipidemia and CVD that are accompanied by elevated levels of CRP.

## INTRODUCTION

Despite the existing advanced lipid-lowering drugs and high-technology invasive methods for their diagnosis and treatment, cardiovascular diseases (CVDs) stubbornly remain the leading cause of death in developed countries. Lipid metabolism disorders are the main factors behind the development and progression of the atherosclerosis underlying CVD, while the C-reactive protein (CRP) is a marker of systemic inflammation. Available data increasingly suggest that CRP is not only an inflammatory marker, but that it can be also regarded as one of the pathogenic components of CVD [1]. The monomeric form of CRP (mCRP) originates from a dissociation of the native pentameric form (nCRP) on the surface of activated platelets and damaged cells [2, 3] and can be found in the necrotic zones after acute myocardial infarction and in atherosclerotic plaques [4]. The high CRP level detected after a myocardial infarction is associated with the risk of later myocardial dysfunction and heart failure [5, 6]. The plasma concentration of CRP is related to the prognosis of disease progression in atherosclerosis, chronic heart failure, atrial fibrillation, myocarditis, aortic regurgitation, and the prognosis after heart transplantation [7]. The CANTOS trial, which involves high-risk patients with an elevated nCRP level (median, 4.1 mg/L), has reliably shown that inflammation suppression without any effect on the low-density lipoprotein cholesterol (LDL-C) concentration significantly reduces the risk of cardiovascular complications, thereby being a new therapeutic strategy for cardiovascular patients [8].

The link between an elevated nCRP concentration and atherogenic apoB-100 containing lipoproteins was demonstrated in several studies. Elevated levels of nCRP and oxidized low-density lipoproteins (oxLDLs) were found in patients with CAD. It was established in an augmentation of atherosclerosis severity that is estimated by the number of affected coronary arteries [9]. High levels of nCRP and lipoprotein(a) (Lp(a)) were observed in a group of patients younger than 45 years with a history of myocardial infarction [10]. M. Gronholdt et al. established that an elevated concentration of acute inflammatory markers is strongly related to an elevated level of triglyceride-rich lipoprotein particles, a larger volume of atheroma, and a higher echogenicity of the plaques located in the carotid arteries, an indication of the role of inflammatory markers as possible predictors of lesion severity and formation of an unstable atherosclerotic plaque [11].

The capabilities to pharmacologically correct the CRP level are currently confined to drugs that affect its synthesis in the liver [12]; meanwhile, means to directly influence the concentration of this protein are being actively sought. Elimination of CRP from a patient’s bloodstream using the extracorporeal methods of therapeutic apheresis is one of the potential solutions. The methods based on adsorption technologies are considered the most effective and selective. Active ingredients of adsorption columns include specific antibodies or synthetic mimetics of natural ligands and the binding sites of CRP molecules.

CRP elimination significantly reduced the necrotic zone in animal models of acute myocardial infarction [13]. A PentraSorb CRP column (Pentracor, Germany) applied in patients with acute myocardial infarction showed a CRP level decreased by 50% within a single procedure [14]. Trials to collect data on the clinical efficacy of such procedures are currently underway.

We have previously elaborated a sorbent containing a synthetic mimetic ligand capable of simultaneously binding CRP and atherogenic lipoproteins [15]. Synchronous reduction of the concentration in the bloodstream of these components allows one to reduce their proinflammatory and proatherogenic activity, and, thereby, influence both major components of the pathogenesis of atherosclerosis.

The aim of this study was to investigate the efficiency and selectivity of the binding proinflammatory and proatherogenic components of human blood plasma using the synthesized sorbent.

## MATERIALS AND METHODS

The research was conducted using purified solutions of low-density lipoproteins (LDL), human serum albumin (HSA), immunoglobulin G (IgG), CRP, and blood plasma or serum (to be more specific, plasma from healthy volunteers stabilized with citrate phosphate dextrose anticoagulant; heparin-containing plasma obtained after plasma exchange; and the serum of patients with CAD). LDL solutions with total cholesterol (TC) concentrations of 500 and 800 mg/dL were obtained from the blood plasma of healthy individuals by ultracentrifugation in the neutral NaBr density gradient [16]. A HSA solution (29 mg/mL) was prepared using a lyophilized sample (Calbiochem, United States). The CRP solution (1 mg/mL) contained 1% HSA as a stabilizer (Imtek, Russia). The IgG solution was a sample of human IgG for intravenous administration (Octapharm, Switzerland) with a concentration of 50 mg/mL.

A comparative analysis was performed using immune sorbents with immobilized polyclonal antibodies against LDL (LDL–Lipopak®), against IgG (Ig Adsopak®, both LTD sorbents manufactured by POCARD, Russia), and against nCRP. The sorbent with a synthetic ligand was obtained by immobilization of aromatic aldehyde on a cross-linked agarose matrix using a molecular spacer according to the method described earlier [17], albeit modified. The synthesis was carried out without glutaraldehyde.

Batch chromatography was applied in all chromatographic studies at room temperature with a 1 : 10 volume ratio between the sorbent gel and the studied sample (i.e., protein solution, plasma, or serum), unless agreed otherwise. To construct the adsorption isotherms, chromatography was performed in a buffer solution containing 10 mM NaH_2_PO_4_, 140 mM NaCl (pH 7.0) for 1 h. The maximum adsorption capacity (S_max_) and desorption constant (K_d_) were calculated according to the isotherms. Permanent load and a plasma dilution from 1 to 5 times were used to estimate the distribution of plasma components during chromatography, which is characterized by the ratio between the concentrations of substances bound to the sorbent and free ones, or the distribution coefficient (K_c_). Chromatography with the concentrated LDL solutions (300–500 mg/dL) was performed to determine the maximum LDL-binding capacity; the amount of free cholesterol was controlled for a period extending from 30 min to 20 h.

The plasma levels of TC, high-density lipoprotein cholesterol (HDL-C), HSA, and triglycerides (TG) were measured using kits manufactured by Analiticon Biotechnologies AG (Germany) and Vector Best (Russia). The IgG and HSA concentrations in the solutions were determined by spectrophotometric methods using molar extinction coefficients of 1.4 and 0.6, respectively. Enzyme immunoassay (Vector Best, Russia) was applied to measure the CRP and IgG concentrations. TheLp(a) concentration was measured using monospecific sheep polyclonal antibodies against human Lp(a) [18]. The LDL-C concentration was calculated using the Friedewald formula: LDL-C = TC – HDL-C – TG/5 [19]. The concentration of the corrected LDL-C (LDL-C_corr_) that allows for a concentration of Lp(a) cholesterol (Lp(a)-C) was calculated using the Friedewald formula with Dahlen’s modification: LDL-Ccorr = LDL-C – 0.33 × Lp(a), where Lp(a) is the Lp(a) concentration in mg/dL [20].

The adsorptions of CRP, IgG, HSA, and LDL onto the sorbent are adequately described with the Langmuir equation: S = S_max_ × [C_sol_]/(K_d_+[C_sol_]), where S is the amount of bound component, Smax is the maximum adsorption capacity, [C_sol_] is the concentration of free component in the solution, and K_d_ is the desorption constant. When recalculating K_d_ into the desorption constants expressed as a particle count (K_d_ M), we used the corresponding molecular weights. Recalculation of the LDL-C concentration into the LDL concentration was performed considering the percentage of cholesterol in the corresponding lipoprotein.

## RESULTS AND DISCUSSION

The synthesized sorbent is a polymeric agarose matrix with a synthetic ligand containing an aromatic group covalently attached via a molecular spacer. The sorbent is characterized by a significantly specific surface and well-developed pores available for all the plasma components studied in this work. The granule size of the matrix varies from 40 to 180 μm; the pore size – or the limit of molecular weight exclusion – is 6.3 × 10^5^ kDa [21].

**Fig. 1:**
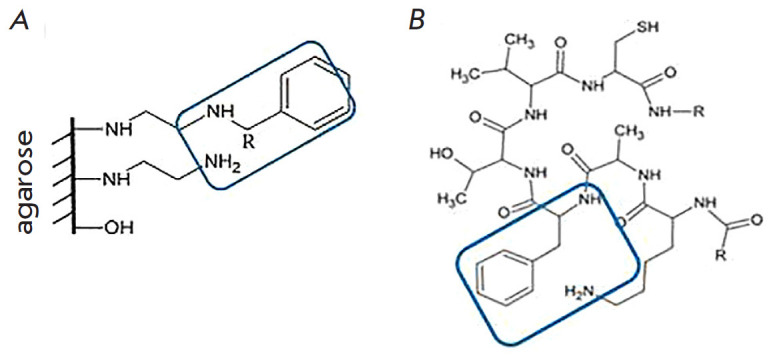
Structural components of the synthesized sorbent (*A*) and the epitope of CRP (**AFTV**) for binding to leukocyte receptor (*B*) [22]

Hydroxyl radicals of monosaccharide agarose residuals, primary and secondary amine groups of the spacer, and phenyl groups of the ligand are the functional groups possible on the surface of the synthesized sorbent (Fig. 1A). Adsorption of plasma components can be performed using an ion exchange, aromatic, and hydrophobic interactions.

When studying peptides that inhibit interaction between the CRP and U937 cell lines, Q. Zen et al found that TKPLK**AFTV**CLH amino acids are of critical significance for the interaction between CRP and the CRP-binding site of the receptor to take place [22]. This sequence contains a section with three hydrophobic amino acids, including a single aromatic group (Fig. 1B). An assumption was made that the sorbent would bind to the ligand according to the principle of complementarity and hydrophobic interactions. Since an investigation of its properties revealed an ability to sorb atherogenic lipoproteins, it is not unlikely that the designed sorbent is a LOX-1 receptor mimetic [23].

**Table 1:** The parameters of adsorption isotherms of human blood plasma proteins such as CRP, IgG, and HSA

	Plasma proteins		
	CRP	IgG	HSA
Molecular weight, kDa	115	146	64
Adsorption characteristics			
Desorption constant, K^d^, M			
Synthetic sorbent	4.2 × 10^-8^	2.9 × 10^-5^	1.4 × 10^-5^
Immunosorbent^*^	1.3 × 10^-8^	7.5 × 10^-7^	um^#^
Adsorption capacity, S^max^, mg/mL of gel			
Synthetic sorbent	34.4	45.2	46.6
Immunosorbent^*^	0.9	16.1	um^#^

^*^ - the sorbent with an immobilized sulfate fraction of polyclonal goat antibodies against human nCRP was used for CRP adsorption;

IgG – Adsopak® sorbent with immobilized polyclonal sheep antibodies against human IgG was used for IgG adsorption;

^#^ - um – unmeasured.

Table 1 shows the adsorption characteristics, and the maximum adsorption capacity (S_max_) and desorption constant (K_d_) in particular, in comparison with the corresponding parameters of the IgG–Adsopak® immunosorbent and the sorbent with immobilized polyclonal antibodies against human nCRP. The desorption constant of 4.2 × 10^-8^ M is an indication of the specific binding of the synthetic sorbent to CRP, suggesting that the functional groups of sorbents can act as mimetics of the CRP-binding site. Interaction between the synthetic sorbent and major protein components of the human blood plasma such as IgG and HSA is substantially less specific (K_d_ for IgG and HSA were 2.9 × 10^-5^ and 1.4 × 10^-5^ M, respectively). Interaction with LDL is characterized by a desorption constant of (7.7 ± 3.6) × 10^-7^ M, similar to that for the LDL–Lipopak immunosorbent ((8.0 ± 2.2) × 10^-7^ M). The large amount of active functional groups in the synthetic sorbent results in the high S_max_ values seen in in vitro experiments with blood plasma or serum.

Plasma lipoproteins serve as ligands for various receptors of the endothelial and smooth muscle surface, as well as for macrophages and platelets. These numerous receptors, capable of binding native and modified LDL, participate not only in cholesterol transport, but also in multiple physiological and pathophysiological processes, including inflammation, repair, and atherosclerosis [24]. Scavenger receptors can bind to a wide range of ligands, including apoB-100 containing modified lipoproteins, Lp(a), and CRP [25, 26], suggesting possible common epitopes for the interaction.

Lp(a) is an LDL-like particle where the apoB-100 molecule is covalently bound to a high-molecular-weight glycosylated apoprotein (a). Although Lp(a) is an independent genetic risk factor of various CVDs, there are no ways to manage it pharmacologically [27, 28]. Therapeutic antisense oligonucleotides designed for these purposes are currently undergoing clinical trials [29].

**Table 2:** The distribution coefficients (K_c_) of human the blood plasma components Lp_(a)_, TG, and HSA

Sorbent	Distribution coefficients (K_c_)		
	Lp(_a_)	TG	HSA
Synthetic sorbent	38 ± 7	7 ± 1	6 ± 5
LDL–Lipopak®	23 ± 6	6 ± 1	5 ± 4

**Fig. 2:**
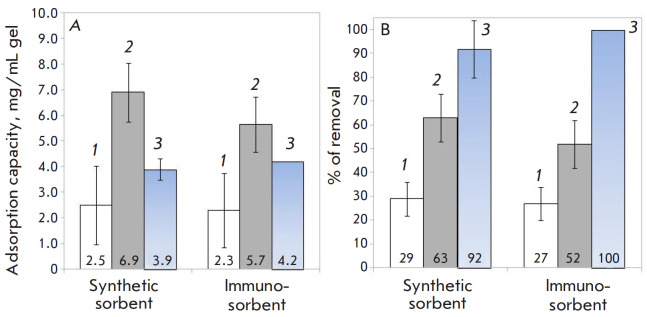
The values of (*A*) adsorption capacity, (*B*) adsorption efficiency (% of removal) of the studied plasma components for the synthetic sorbent and the LDL–Lipopak® immunosorbent. The studied plasma components: (*1*) TG, (*2*) Lp(a), and (*3*) LDL-C_corr_. The initial plasma concentrations were 149 mg/dL for TC, 48 mg/dL for HDL-C, 108 mg/dL for TG, 109 mg/dL for Lp(a), and 42 mg/dL for LDL-C_corr_ (the estimated value)

The adsorption of Lp(a), TG, and LDL-C_corr_ was investigated using chromatography of human blood plasma with varied concentrations of the studied components (dilution 1 to 5 times) and a permanent load of 1 mL of plasma per 0.1 mL of synthetic or immune sorbents. The distribution coefficients (K_c_) are shown in Table 2; adsorption capacity and adsorption efficacy (% of removal) are shown in Fig. 2. Adsorption of Lp(a) was more pronounced compared to that of TG and HSA; the largest differences in the Kc values were recorded for the synthetic sorbent. The LDL–Lipopak immune sorbent was characterized by a better interaction with LDL, as shown by high values of the adsorption efficacy.

Adsorption of CRP was studied in vitro in a human blood serum with an extremely high CRP concentration (1330 mg/L). The specificity of the interaction between the synthetic sorbent and CRP was convincingly proved by a high binding efficiency (101), while HSA and IgG were characterized by a K_c_ value amounting to 2. Based on the results of this experiment, the adsorption capacity of the synthetic sorbent reached 12 mg of CRP per mL of gel.

**Fig. 3:**
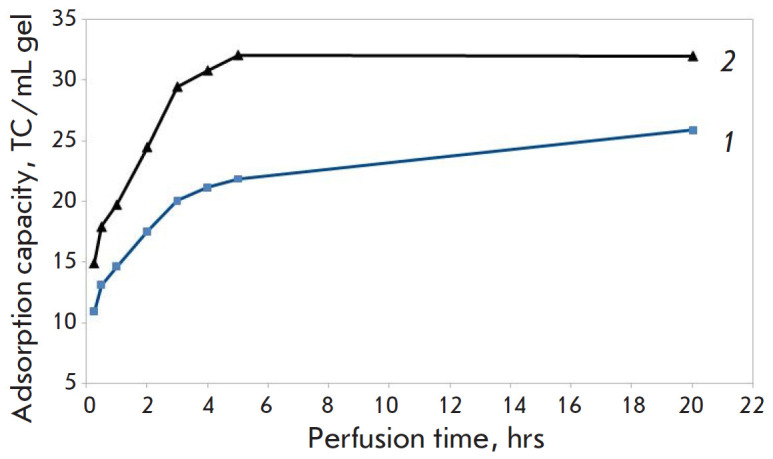
Adsorption capacity of the synthetic as a function of load and chromatography duration (from 30 min to 20 h). *1 *– 30 mg of TC per mL of gel, *2 *– 50 mg of TC per mL of gel

Incubation of the synthetic sorbent with concentrated LDL solutions (300 and 500 mg/dL) for a long period of time showed that maximum LDL-binding capacity was not attained until the 6^th^ hour of incubation, which was apparently associated with the steric peculiarities of the interaction between the active functional groups of the sorbents and such a large supramolecular complex as LDL. The adsorption capacity after 6 h of incubation with a higher load was 32 mg of TC per mL of gel, with 64% binding efficiency. The adsorption capacity was lower (26 mg of TC per mL of gel) under a lower load after 20 h of incubation, although the binding efficiency appeared to be higher (86%). Figure 3 shows the adsorption capacity of the synthetic sorbent as a function of the load and chromatography duration.

## CONCLUSIONS

Our experimental data point to a high specificity of the interaction between the synthetic sorbent and CRP. The desorption constant (K_d_) (4.2 × 10^-8^ M) was 1000 times higher than that of major plasma proteins, such as HSA and IgG, while the distribution coefficient (K_c_), equal to 101, was 50 times higher than those of HSA and IgG. The ability to bind to the majority of atherogenic lipoproteins was demonstrated; the maximal adsorption capacity in a LDL solution is 32 mg of TC per mL of gel sorbent. This sorbent can be recommended for a complex elimination of CRP and atherogenic lipoproteins from the blood plasma of patients with refractory hyperlipidemia and CVD that are accompanied by elevated CRP levels.

## LIMITATIONS

The adsorption isotherm of CRP was constructed using a solution containing CRP (1 mg/mL) and HSA (10 mg/mL). The distribution coefficient of LDL-C (LDL-Ccorr) was not calculated because of the low initial LDL concentration in the blood plasma. The duration of the chromatography (1 h) was shorter than that required for the saturation of the synthetic sorbent at high LDL concentrations.

## Abbreviations

CRP: C-reactive protein
mCRP: the monomeric form of CRP
nCRP: the pentameric form of CRP
Lp(a): lipoprotein(a)
apoB-100: apolipoprotein B-100
LDL: low-density lipoprotein
HDL: high-density lipoprotein
oxLDL: oxidized LDL
TC: total cholesterol
TG: triglyceride
HSA: human serum albumin
IgG: immunoglobulin G
LDL-C: low-density lipoprotein cholesterol
HDL-C: high-density lipoprotein cholesterol
CVD: cardiovascular diseases
